# Midwives’ oral health recommendations for pregnant women, infants and young children: results of a nationwide survey in Germany

**DOI:** 10.1186/s12903-016-0192-1

**Published:** 2016-03-18

**Authors:** Yvonne Wagner, Roswitha Heinrich-Weltzien

**Affiliations:** Department of Preventive Dentistry and Pediatric Dentistry, Jena University Hospital, Bachstr. 18, 07743 Jena, Germany

**Keywords:** Oral health, Prevention, Tooth brushing

## Abstract

**Background:**

Studies suggest that poor oral health during pregnancy can lead to perinatal complications, such as low birth weight and preterm delivery as well as poor oral health in children. Aim of this study was to assess the German midwives knowledge about oral health and preventive recommendations for pregnant women, infants and young children.

**Methods:**

The nationwide online-survey was conducted with use of a self-developed, pretested and validated standardized questionnaire. The German association of midwives (Deutscher Hebammenverband e.V.) informed their members about the survey through email, newsletter, website and association journal (Hebammenforum) (*n* = 7.500). Data were analyzed using descriptive statistics.

**Results:**

Response rate was 12.6 % (mean age 42.9 ± 9.3 years). The majority of midwives advised pregnant women about periodontal diseases (78.6 %). Of the midwives, 8.4 % mentioned the possible associations between periodontal diseases and perinatal complications. In general, half of the midwives (53.5 %) recommended a dental visit during pregnancy. A total of 65.5 % of midwives advised parents about early childhood caries. The majority of midwives recommended that oral hygiene starts with eruption of the first tooth (60.4 %) and the first dental visit of the child should be at age 2 or 3 years (51.6 %). Midwives recommendations regarding the implementation of oral hygiene and the referral to a dentist during pregnancy and childhood were highly variable.

**Conclusions:**

To increase oral awareness and to improve the oral health knowledge among midwives and all other health-care professionals, uniform guidelines should be developed in Germany.

**Trial registration:**

German Clinical Trial Register DRKS00008021

## Background

Recently there has been increased awareness of the role of maternal oral health and its potential impact on the child [1–3]. During pregnancy, dietary and hormonal changes, as well as increased nausea and vomiting, can affect the dental and the gingival tissues, inducing microbiological changes in the oral flora and immunosuppression [1–3]. Studies suggests that poor oral health during pregnancy can lead to perinatal complications, such as low birth weight and preterm delivery as well as poor oral health in children [1–4]. While some studies demonstrated an association between periodontal disease and perinatal complications, the causal evidence is not conclusive [5–8].

Oral health is a key to overall health and well-being, nevertheless, during pregnancy it is often neglected by women, particularly among women of low socioeconomic status [1, 3, 4, 9]. A history of cavities or active caries in mothers is a predictor for early childhood caries (ECC) [10]. ECC is a major global public health issue and the most common chronic disease in children [10–13]. It affects 5–94 % of 1- to 5-year-old children worldwide [11, 12]. Children suffer from dental pain, and difficulties with eating, speaking and socialising [10–13]. In addition, children with ECC can have tooth developmental disorders, eruption problems and a high risk of caries in permanent dentition [10–13].

Against this background, pregnant women are recommended to visit the dentist during pregnancy, and to maintain good oral hygiene with daily tooth brushing and the use of fluoride toothpaste. Children are recommended to visit the dentist in their first year of life, and to start daily tooth brushing and using sufficient fluoride on eruption of the first tooth [4, 9–17].

In Germany, paediatricians and dentists disagree about the use of fluoride supplements and fluoride-containing toothpaste for children aged 0–3 years [18, 19]. The German Society of Oral and Maxillofacial Surgery agrees with other societies, like the European Academy of Paediatric Dentistry and the American Academy of Paediatric Dentistry, and prefers that tooth brushing should start with fluoride toothpaste when the first tooth erupts and the use of vitamin D supplements immediately after birth [11, 16–18]. The German Society of Paediatrics and Adolescent Medicine and the German Academy for Paediatrics and Adolescent Medicine suggest that fluoride supplements (vitamin D and fluoride supplements in combination) should be prescribed immediately after birth and that a child should start using toothpaste by 4 years of age if they can spit it out [19]. This disagreement over the preventive regime in the first 3 years of life leads to confusion about the benefit of fluoride in parents, primary health-care providers, paediatricians, dentists and midwives.

In Germany, maternal oral health education is anchored in the maternity policy guidelines [20]. Gynaecologists should inform and emphasise the importance of oral health for both mother and child [20]. However, health professionals often do not provide oral health care to pregnant women [4, 9, 10, 15]. The majority of pregnant women received no information about oral health care during pregnancy, and much less than half of pregnant women consult a dentist [4, 5, 14, 21]. Misunderstandings and beliefs that pregnancy per se has an adverse effect on the teeth and periodontal tissues or the fears of undergoing dental treatment, radiographs, extractions and local anaesthesia during pregnancy are aggravating factors and increase the lack of access [4, 9, 14, 21, 22]. Antenatal care providers are now recommended to promote maternal oral health [10, 11, 14, 15]. Unlike dentists, midwives are often the first health professional pregnant women encounter and they support families in the child’s first year. Therefore, midwives are the preferred health-care professionals for passing on key health messages to pregnant women, for sensitising them about oral health and for referring them to a dentist [4, 9, 10, 14–16]. Some countries already use this new approach and have implemented preventive strategies to maintain maternal oral health during pregnancy [14–16, 23, 24]. To develop such a service in Germany, it is necessary to expand the scope of practice of midwives, and to enable them to acquire new skills and knowledge in this important area. First, the focus should be the identification of the educational needs of midwives and to develop appropriate educational material.

Considering this background, we wanted to assess midwives’ knowledge of oral health and preventive recommendations for pregnant women, infants and young children, and which guideline they follow (paediatric or dentistry society guidelines) through a nationwide online survey in Germany. As a secondary objective of this study, we wanted to examine whether there are differences between midwives in terms of oral health practices after taking into account their characteristics (age, professional experience, working condition and training).

## Methods

### Study population

The nationwide survey was conducted online using the software package SoSci Survey (SoSci Survey GmbH, Munich, Germany). Participants were non-retired midwives currently practising in Germany. The study population was identified through the database of the German Association of Midwives (Deutscher Hebammenverband e.V.). Midwives were informed in four ways about the survey (by email, a newsletter, the web site and association journal (*Hebammenforum*), so that a total of 7,500 midwives could be reached. No incentives were offered. The survey started on February 1, 2015 and was closed on April 30, 2015. The Ethics Committee of Jena University Hospital approved this study (3941-12/13; DRKS 00008021).

### Questionnaire

The survey instrument was a 16-item standardised questionnaire that took approximately 10 min to complete. It included closed questions with predetermined answers and open-ended questions. The open questions allowed a differentiated analysis. The actual wording of the survey questions and response options are presented in Table 1.

**Table 1:** Questionnaire with survey questions and response options (multiple responses possible)

Survey questions	Response options
Age	
Work experience	
Working condition (multiple responses possible)	Private practice Employed Freelance Education and training
Do you advise pregnant women and parents about oral health? On which topics?	
Do you recommend a dental visit during pregnancy?	Yes No
What do you recommend regarding oral hygiene for pregnant women?	
What do you recommend regarding oral hygiene for infants?	
When do you recommend tooth brushing should start for infants? (multiple responses possible)	With the first tooth First birthday Second birthday Third birthday Fourth birthday Later
When do you recommend the first dental visit for infants should take place?	
Do you recommend the use of vitamin and/or fluoride supplements? (multiple responses possible) Please specify the beginning and the end of consumption.	Vitamin D Vitamin D combined with fluoride Fluoride
Do you advise pregnant women about periodontal diseases during pregnancy? Please specify.	
Do you advise parents about early childhood caries? Please specify.	
Was oral health a part of your midwifery education?	Yes No
Have you ever been to advanced training on oral health for pregnant women and infants?	Yes No
Which resources do you use for information?	
Are you interested in further education and training on oral health?	Yes No
Comments	

The questionnaire was developed, pretested and validated in cooperation with midwives in the German Association of Midwives, a university of applied sciences (department of health and nursing) and a maternity ward. To develop the structured guideline for the standardised questionnaire, interviews (a fixed sequence of questions, but completely open) with three midwives (one in a private practice, one employee and one in education and training) were conducted and evaluated. After that, the developed questionnaire was tested regarding face validity and content validity using respondents (midwives of the maternity ward) and a panel of experts (midwives in the German Association of Midwives, and a university of applied sciences). The revised questionnaire was then tested in a pilot test by collecting data from 20 midwives not included in the final sample.

### Data analysis

Data were recorded in Excel files and transferred to the Statistical Package for Social Sciences (SPSS version 20, IBM Corporation, Armonk, NY, USA). Data were analysed using descriptive statistics. The analysis of the open questions was performed with a summarised content analysis. Therefore, categories and keywords were defined (for example, category “oral hygiene” and keywords “tooth brushing”, “toothpaste” and “fluoride”) during the development and validation of the questionnaire. The final responses were filtered using a computer by a keyword search and sorted according to the frequencies. Midwives’ responses regarding their age, professional experience, working condition and training were compared using a chi-square test (Pearson). Differences were considered statistically significant for *p* < 0.05.

## Results

The response rate was 12.6 % (947 participants). The mean age was 42.9 ± 9.3 years with an average professional experience of 18.7 ± 9.2 years. Table 2 presents the results of the questionnaire.

**Table 2:** Midwives’ preventive recommendations and results of the questionnaire (947 participants)

Survey questions	Results
Age	42.9 ± 9.3 years 4.1 % Non-response
Work experience	18.7 ± 9.2 years 0.0 % Non-response
Working condition	11.2 % Private practice 12.2 % Employed 23.2 % Freelance 51.5 % Employed and freelance 1.9 % Education and training system 0.0 % Non-response
Do you recommend a dental visit during pregnancy?	53.5 % Yes 43.5 % No 3.0 % Non-response
When do you recommend tooth brushing should start for infants?	60.4 % With the first tooth 22.8 % First birthday 11.5 % Later 5.3 % No specification 0.0 % Non-response
When do you recommend the first dental visit for infants should take place?	6.8 % In first year of life 51.6 % At age 2 to 3 years 10.1 % Referred to mother’s dental visit 31.5 % No specification 0.0 % Non-response
Do you advise pregnant women about periodontal diseases during pregnancy?	78.6 % Yes 19.3 % No 2.1 % Non-response
Do you advise parents about early childhood caries?	65.5 % Yes 32.0 % No 2.5 % Non-response
Have you ever been to advanced training on oral health for pregnant women and infants?	9.7 % Yes 90.3 % No 0.0 % Non-response
Are you interested in further education and training on oral health?	86.7 % Yes 13.3 % No 0.0 % Non-response

### Responses to closed questions

The majority of midwives advised pregnant women about periodontal diseases (78.6 %). In general, half of the midwives (53.5 %) recommended a dental visit during pregnancy. A total of 65.5 % of midwives advised parents about ECC. The majority of midwives recommended that oral hygiene starts with eruption of the first tooth (60.4 %). Most of the midwives (65.1 %) recommended the use of vitamin D, vitamin D combined with fluoride and fluoride supplements (Tables 3 and 4). The recommendation of fluoride supplements was rejected by 18.3 % of midwives.

**Table 3:** Midwives’ preventive recommendations regarding vitamin and fluoride supplements

Recommendation of supplements			Midwives (%)
Vitamin D	Vitamin D combined with fluoride	Fluoride tablets	
Yes	No	No	18.3
No	Yes	No	0.6
Yes	Yes	Yes	65.1
Yes	Yes	No	14.7
No	Yes	Yes	0.5
Yes	No	Yes	0.8

**Table 4:** Midwives’ preventive recommendations regarding vitamin and fluoride supplements

Recommendation of period of use of Vitamin D supplements		Midwives (%)
Beginning of consumption	End of consumption	
First month of life	Second year of life	100.0

The preventive recommendations were given regardless of the age, professional experience or working conditions of the midwives (Table 5). The answers of midwives who had already received advanced training in oral health were significantly different regarding the counselling about periodontal disease and ECC (*p* < 0.001), recommendations for the referral to a dentist during pregnancy (*p* < 0.001), the recommended age for the first dental visit (*p* < 0.001), and the timing of commencement of tooth brushing (*p* < 0.001) (Table 5).

**Table 5:** Midwives’ preventive recommendations depending on age, working condition and advanced training in oral health (proportions in per cent)

Recommendations		Age		*p*-value	Working condition					*p*-value	Advanced training		*p*-value
		<40 years	>40 years		Private practice	Employee	Freelance	Employee and freelance	Education and training system		Yes	No	
		(*n* = 373)	(*n* = 574)		(*n* = 106)	(*n* = 116)	(*n* = 220)	(*n* = 487)	(*n* = 18)		(*n* = 92)	(*n* = 855)	
Consulting about periodontal disease	Yes	78.3	78.9	0.816	78.2	78.3	78.6	78.6	78.8	0.999	94.6	75.4	<0.001
	No	21.7	21.1		21.8	21.7	21.4	21.4	21.2		5.4	24.6	
Consulting about early childhood caries	Yes	65.2	65.8	0.823	65.1	65.8	65.5	65.6	65.5	0.999	97.8	63.2	<0.001
	No	34.8	34.2		34.9	34.2	34.5	34.4	34.5		2.2	36.8	
Dental visit during pregnancy	Yes	53.1	53.9	0.821	53.1	53.6	53.2	53.7	53.5	0.874	96.7	49.2	<0.001
	No	46.9	46.1		46.9	46.4	46.8	46.3	46.5		3.3	50.8	
Child 1st dental visit	1st year of life	6.3	7.3	0.530	6.6	6.8	6.4	6.9	6.8	0.439	98.5	6.2	<0.001
	2nd/3rd birthday	51.3	51.9		51.9	51.9	51.8	51.6	51.1		0.0	51.9	
	Mother’s dental visit	11.5	8.7		31.4	30.3	31.8	31.6	31.3		1.5	10.0	
	No specification	30.9	32.1		10.1	11.2	10.0	9.9	10.8		0.0	31.9	
Toothbrushing	With 1st tooth	60.1	60.7	0.879	59.9	60.3	60.5	60.6	60.5	0.999	98.5	59.9	<0.001
	With 1st birthday	22.7	22.9		23.0	22.5	23.6	22.5	22.7		1.5	22.8	
	Later	11.3	11.7		11.0	11.1	11.8	11.4	11.5		0.0	11.8	
	No specification	5.9	4.7		6.1	6.0	4.1	5.5	5.3		0.0	5.5	

Chi-square test (Pearson) was used to compare groups

*p* < 0.05, statistically significant, Chi-square test

### Responses to open-ended questions

Counselling about periodontal diseases during pregnancy included gum swelling and bleeding and the importance of good daily oral hygiene with the use of fluoride toothpaste. Of the midwives, 8.4 % mentioned the possible associations between periodontal diseases and perinatal complications. Midwives advice about ECC included the possible consequences of the prolonged use of the bottle and sweetened beverages. Furthermore, 26.8 % offered advice about the frequency of tooth brushing, and 1.3 % about the dosage of toothpaste. Fluoride toothpaste was recommended by 43.1 %. Most midwives (51.6 %) recommended that the first dental visit of the child should be at age 2 or 3 years. One-third (31.5 %) of the midwives gave no recommendations; 10.1 % referred to the mother’s dental visit and 6.8 % of the midwives recommended the first dental visit should be in the first year of life. All midwives recommended the period of use of vitamin D supplements according to the paediatric guideline as being from the first month of life to the second year of life (Tables 3 and 4).

## Discussion

This study showed that more than half of the midwives used a mix of the current paediatric and dentistry guidelines; they emphasise the importance of daily oral hygiene and the use of fluoride toothpaste during pregnancy and they counsel the parents about the possible consequences of the prolonged use of the bottle and sweetened beverages. However, knowledge regarding the association between poor maternal oral health and low birth weight and preterm delivery was limited, and recommendations regarding the implementation of oral hygiene and the referral to a dentist during pregnancy and childhood were highly variable.

This study was based on data from a nationwide online survey in Germany. A comparison with nationally and internationally conducted surveys among midwives showed similar results [4, 10, 19–21]. Midwives were familiar with recommendations about caries prophylaxis, but only few gave information about gingival changes during pregnancy [19–21]. Most midwives were unaware of the possible ill effects on maternal and child health [19–21].

Even gynaecologists and physicians did not routinely advise women about oral health during pregnancy [4, 5, 10, 15, 22]. A survey among physicians showed that only half of them agreed that oral health could affect the outcomes of pregnancy [22]. There is a strong need to increase awareness among health-care professionals about the changes that can occur in the oral cavity during pregnancy and the importance of oral health care [1, 3, 10–12, 15, 16, 19–22]. Additionally, the study findings showed uncertainty and wide variability regarding the implementation of oral hygiene and the referral to a dentist. The majority of midwives (60.4 %) recommended that oral hygiene starts with the eruption of the first tooth. The additional recommendation of the use of fluoride toothpaste was mentioned by 43 % of the midwives. Most midwives recommended the first dental visit should take place after the second or third birthday (51.6 %), and one-third gave no recommendations. These results reflect the current recommendations of the health insurance system [18–20]. In Germany the health insurance providers recommend that the first dental visit should take place between 30 and 42 months of age [18–20]. Internationally, there is a demand for early establishment of regular dental care by age one and the start of tooth brushing with the first tooth [6–9, 11, 13–15, 24]. Unfortunately, most paediatricians also refer children to a dentist at the age of 3 and recommend that tooth brushing and use of toothpaste start after the first birthday [23]. The disagreement and the different viewpoints about the recommendation of the first dental visit, the preference for topical fluoride application by tooth brushing versus systemic fluoridation by tablets between the German societies is one possible explanation for the uncertainty among midwives [24, 25]. The majority of midwives followed both guidelines, and recommended that the first dental visit should take place between 2 and 3 years, and recommended the use of vitamin D, vitamin D combined with fluoride and fluoride supplements [24, 25]. The results revealed that there is a strong need for the development of uniform guidelines that are based on agreement between the societies of paediatricians and the societies of paediatric dentists in Germany. This is also confirmed by the results of other studies in Germany, which showed that the implemented oral health recommendations among paediatricians and dentists were also heterogeneous [23, 26].

As a secondary objective of this study, we wanted to examine whether there were differences among midwives in terms of oral health practices when taking their characteristics into account. This study found that the preventive recommendations were given irrespective of the age, professional experience or working conditions of the midwives. The preventive recommendations of midwives who had already received advanced training in oral health were significantly different from their colleagues. Trained midwives were aware of the role of maternal oral health and its potential impact on the child. Midwives who had received advanced training counselled the women about periodontal disease, ECC, the importance of a dental visit during pregnancy and for the child in the first year of life, and the timing of commencement of tooth brushing with the first tooth more often than midwives with no training.

Midwives play an important role in healthy growth and can provide anticipatory guidance and improve oral health awareness [10–12, 17–21]. Up to now, oral health has played no or a minor role in the German midwifery curriculum, and there have been no special oral health training programmes for midwives in Germany. To improve the oral health of pregnant women and children, oral health training courses should encourage midwives to counsel pregnant women and parents about the importance of oral health care. The majority of midwives (86.7 %) in this study were interested in further education and training on oral health, but highlighted barriers, such as the time and remuneration, as well as the need for uniform recommendations for dentists and paediatricians regarding fluoride prophylaxis. Cooperation between dental and other health professionals, like midwives, gynaecologists and paediatricians, may be a successful approach to increase dental awareness among caregivers [4, 10–12].

Some limitations of this study need to be addressed. This survey was conducted nationally; nevertheless, the results were limited to those midwives who participated. For the evaluation and generalisability of the results, a comparison was made with other studies of midwives. These had similar results and confirmed the study findings [4, 10, 19–21]. A second limitation of this study was the response rate. On the one hand, the content of a study called “Midwives’ oral health recommendations” is outside what midwives are concerned about, especially against the background of their own existential problems in Germany. On the other hand, it is an internationally increasingly difficult problem to achieve high return rates [27, 28]. Response rates in web surveys vary from 0 to 100 %, depending on the topic, response method and level of incentives [27, 28]. Previous German surveys among midwives had 264 to 3603 participants [21, 29, 30]. To improve the response rate of our survey, midwives were informed about the survey through emails, a newsletter, the web site and the association journal. In this way, we were able to achieve a response rate of 12.6 %. Higher rates could have been achieved with more effort and higher costs, but the cost–benefit ratio was not justified for this study. Of course, a response rate of about 13 % means that approximately 87 % of the midwives did not respond and that there could be potential bias. A meta-analysis of the impact of non-response rates on the non-response bias of 566 standard estimates from 44 studies showed that non-response rates could explain 8 to 19 % of the variation in different estimates of the non-response bias [31]. In this study, no analysis of the group of non-responders was made due to cost and effort. A comparison with other studies of midwives and a review of the literature about non-response rates and non-response bias have shown that no other results could be reasonably expected [4, 10, 19–21, 27–32]. In addition, it has to be mentioned that due to the individual varying completeness of the responses, it could be possible that, although the midwives advised pregnant women and parents about oral health, the advice given is incorrect or inaccurate. The use of open-ended questions provided a more detailed analysis of the midwives’ knowlegde and educational needs. Nevertheless, it may be that the responses would have been different had midwives been asked specifically to certain topics.

This was the first nationwide survey examining midwives’ knowledge about oral health and preventive recommendations for pregnant women, infants and young children in Germany. The results revealed that midwives had a basic knowledge of the major oral diseases; however, oral health recommendations regarding the implementation of oral hygiene and the referral to a dentist were highly variable. In summary, it can be stated that a uniform guideline for all health-care professionals has to be developed to increase oral awareness and to improve oral health knowledge.

## Conclusions

To increase oral awareness and to improve the oral health knowledge among midwives and all other health-care professionals, uniform guidelines should be developed in Germany.

## Abbreviations

ECC: early childhood caries
DGZMK: German Society of Oral and Maxillofacial Surgery
EAPD: European Academy of Paediatric Dentistry
AAPD: American Academy of Paediatric Dentistry
DGKJ: German Society of Paediatrics and Adolescent Medicine
DAKJ: German Academy for Paediatrics and Adolescent Medicine
